# Domestic violence and child well-being: parenting self-efficacy as a developmental pathway

**DOI:** 10.3389/fsoc.2026.1771909

**Published:** 2026-09-21

**Authors:** Vanisha L. Brown, Chelsei K. Granderson, Leah R. Alexander, Chau-Kuang Chen, Marya L. Shegog, Shannon Alvarez, April E. Curry, Adrian Dexter Samuels, Leslie Halpern, Pandu Gangula

**Affiliations:** 1 Department of Public Health, School of Global Health, Meharry Medical College, Nashville, TN, United States; 2 Tennessee Department of Health, Nashville, TN, United States; 3 Institutional Research, Meharry Medical College, Nashville, TN, United States; 4 Power Integration Center, Las Vegas, NV, United States; 5 School of Graduate Studies, Meharry Medical College, Nashville, TN, United States; 6 Center for Health Policy, School of Global Health, Meharry Medical College, Nashville, NY, United States; 7 Oral/Maxillofacial Surgery Residency, New York Medical College, Valhalla, NY, United States; 8 Oral Diagnostic Sciences and Research, School of Dentistry, Meharry Medical College, Nashville, TN, United States

**Keywords:** child well-being, domestic violence, family-centered interventions, parenting self-efficacy, trauma-informed care

## Abstract

Domestic violence is a pervasive public health and social issue with consequences that extend beyond survivors to affect caregiver functioning, family relationships, and child development. Children may be affected through direct exposure to violence and through disruptions in the caregiving environment, including caregiver trauma, psychological distress, parenting stress, and reduced emotional availability. This commentary examines a proposed developmental pathway linking domestic violence, parenting self-efficacy, and child well-being. Parenting self-efficacy refers to caregivers’ beliefs in their ability to effectively carry out parenting responsibilities and positively influence their children’s development. Domestic violence may undermine these beliefs by diminishing caregivers’ emotional resources, autonomy, and confidence, potentially affecting parenting practices, parent–child relationships, and children’s cognitive, emotional, behavioral, social, and physical well-being. Conversely, stronger parenting self-efficacy may support responsive caregiving, emotional security, adaptive coping, and family resilience. The commentary identifies parenting self-efficacy as a potentially modifiable target and proposes priorities for research, practice, and family-centered intervention. These priorities include expanding qualitative research on family experiences and parenting processes, strengthening parenting self-efficacy through trauma-informed and family-centered approaches, and developing developmentally responsive support for children. Addressing the interconnected needs of caregivers and children may strengthen family functioning and reduce the long-term consequences of domestic violence for children and families.

## Introduction

Domestic violence (DV) remains a pervasive public health and social issue with far-reaching consequences for individuals, families, and communities. Although the immediate physical and psychological effects on survivors are well documented, the broader impact of DV extends beyond the survivor to influence family functioning, caregiving relationships, and child development. Children living in households affected by DV may experience adverse developmental outcomes through both direct exposure to violence and indirect disruptions in the caregiving environment. Increasing recognition of these interconnected pathways has shifted attention toward understanding the family processes through which domestic violence influences child well-being, particularly the role of parenting in promoting resilience or exacerbating risk (Sousa et al., 2022; Walker-Descartes et al., 2021).

Children’s experiences of domestic violence are heterogeneous and extend beyond witnessing discrete acts of violence. Exposure may include hearing violent incidents, observing injuries or emotional distress, experiencing disruptions in daily routines, intervening during violent episodes, or living with the continuing fear and instability associated with coercive and abusive environments. Such exposure may affect children directly through trauma-related psychological, behavioral, and developmental responses and indirectly through its effects on caregiver mental health and family functioning. Caregivers coping with trauma, depression, anxiety, fear, or diminished emotional resources may encounter difficulty maintaining consistent, responsive, and developmentally supportive parenting practices. Thus, the effects of domestic violence on children should be understood not only as consequences of exposure to violence but also as outcomes shaped by the caregiving relationships and family processes within which development occurs (Arai et al., 2021; Sousa et al., 2022; Walker-Descartes et al., 2021).

Parenting self-efficacy provides a valuable framework for understanding how domestic violence may influence child well-being through disruptions in caregiving processes. It refers to caregivers’ beliefs in their ability to manage parenting responsibilities and foster their children’s healthy development (Jones and Prinz, 2005; Wittkowski et al., 2017). Domestic violence may undermine caregivers’ psychological well-being and confidence, potentially affecting parenting practices, parent–child relationships, and children’s developmental outcomes (Sousa et al., 2022). Examining parenting self-efficacy therefore shifts the discussion beyond documenting the consequences of domestic violence toward understanding a potentially modifiable process through which those consequences may emerge.

This commentary synthesizes current evidence to examine a proposed developmental pathway linking domestic violence, parenting self-efficacy, and child well-being. Specifically, it explores how domestic violence may affect caregiver mental health, parenting confidence, caregiving behaviors, and parent–child relationships, thereby influencing children’s cognitive, emotional, behavioral, social, and physical development. By integrating evidence across these interconnected domains, this commentary identifies parenting self-efficacy as a potentially modifiable target for intervention. It considers implications for research, practice, and family-centered interventions designed to support caregivers and children affected by domestic violence.

### Domestic violence: definition and public health impact

Domestic violence (DV) is a complex social and public health issue encompassing a pattern of abusive behaviors used to establish and maintain power and control within intimate relationships (U.S. Department of Justice, 2025). Although the terms domestic violence and intimate partner violence (IPV) are often used interchangeably, they are not synonymous. IPV refers specifically to physical violence, sexual violence, stalking, or psychological aggression perpetrated by a current or former intimate partner (Centers for Disease Control and Prevention, 2015). Because much of the empirical literature examined in this commentary focuses on violence between intimate partners, the broader term domestic violence is used throughout this commentary while acknowledging that many cited studies specifically investigate IPV. Clarifying this terminology provides a consistent conceptual framework for examining how violence within the home influences parenting processes and child well-being.

Domestic violence represents a substantial global public health burden. Recent estimates indicate that nearly one in three women worldwide has experienced physical or sexual violence during her lifetime, most often perpetrated by an intimate partner (World Health Organization, 2025). In the United States, intimate partner violence affects both women and men, although prevalence, severity, and associated consequences vary across populations (Chen et al., 2023). Sexual minority individuals experience a disproportionate burden; in particular, bisexual women report a higher lifetime prevalence of intimate partner violence than heterosexual women (Chen et al., 2023). Beyond its impact on individuals, domestic violence imposes substantial societal costs, including medical and mental health care, lost productivity, criminal justice involvement, and other social expenditures. The estimated lifetime economic burden of intimate partner violence in the United States approaches $3.6 trillion in 2014 U.S. dollars over victims’ lifetimes (Peterson et al., 2018), emphasizing the need for coordinated prevention and intervention strategies that address its effects on individuals, families, and communities.

### Children’s exposure to domestic violence and child well-being

Children’s exposure to domestic violence is distinct from direct child maltreatment, although the two experiences may co-occur within families (Holt et al., 2008; Pearson et al., 2023). Exposure may include witnessing violent incidents, hearing threats or assaults, observing injuries or property damage, intervening during an altercation, or experiencing the emotional and practical consequences of violence within the household (National Child Traumatic Stress Network, 2015). Children may therefore be affected even when they are not the direct targets of abuse (National Child Traumatic Stress Network, 2015). Exposure may also begin prenatally, as intimate partner violence during pregnancy has been associated with adverse birth outcomes and may influence children’s subsequent development through interconnected biological, psychological, and caregiving pathways (Guo et al., 2023; Kim et al., 2024). Exposure to violence between caregivers can undermine children’s sense of safety and disrupt family routines, relationships, and developmental environments (Arai et al., 2021; Holt et al., 2008). Childhood exposure to domestic violence is therefore an important public health and developmental concern with potential consequences for well-being across the life course (Holt et al., 2008; Vu et al., 2016).

Exposure to domestic violence can adversely affect multiple dimensions of child well-being, including emotional, behavioral, cognitive, social, and physical development (Holt et al., 2008; Howell et al., 2016). Children exposed to violence within the home are at increased risk for anxiety, depression, post-traumatic stress symptoms, behavioral problems, sleep disturbances, impaired academic performance, and difficulties with peer and later interpersonal relationships (Holt et al., 2008; Howell et al., 2016; National Child Traumatic Stress Network, 2015). These effects may persist into adolescence and adulthood, contributing to longer-term difficulties in mental health, behavioral adjustment, and psychosocial functioning (Holt et al., 2008; Vu et al., 2016). However, these outcomes are not inevitable and may be influenced by protective factors, particularly stable and supportive relationships with caring adults and access to appropriate services (Holt et al., 2008; National Child Traumatic Stress Network, 2015). Understanding these developmental consequences provides important context for examining how parenting processes, particularly the strength or erosion of parenting self-efficacy, may buffer or intensify the effects of domestic violence on child well-being.

Children’s outcomes are also shaped by the broader social and family contexts in which domestic violence occurs. Exposure may co-occur with child maltreatment, caregiver mental health or substance-use problems, economic hardship, housing instability, and other forms of adversity, making it difficult to attribute developmental outcomes to a single experience (Pearson et al., 2023; Sousa et al., 2022). Childhood exposure to intimate partner violence has also been associated with an increased risk of involvement in violent intimate relationships during adulthood, including perpetration or victimization; however, such intergenerational patterns are probabilistic rather than inevitable and are influenced by relational, socioeconomic, cultural, and protective factors (Kimber et al., 2018). Recognizing these intersecting conditions reinforces the importance of interpreting children’s outcomes within their broader family and social environments rather than viewing domestic violence exposure as an isolated risk.

The developmental consequences of domestic violence do not occur in isolation but are often shaped by changes in caregiver functioning and family dynamics (Sousa et al., 2022). Domestic violence may compromise caregivers’ emotional well-being, increase parenting stress, and make consistent and responsive parenting more difficult, potentially limiting their capacity to meet children’s emotional, developmental, and behavioral needs (Sousa et al., 2022). These disruptions may, in turn, reduce children’s emotional security, contribute to inconsistent caregiving environments, and increase the risk of adverse emotional and developmental outcomes (Holt et al., 2008; Sousa et al., 2022). Domestic violence may also affect caregiver-child attachment relationships by disrupting the safety, emotional availability, and responsive caregiving through which children develop trust in and seek comfort from their caregivers; however, attachment outcomes vary across children and families and should not be viewed as inevitable consequences of exposure (Noonan and Pilkington, 2020). Consistent with this family-centered perspective, NCTSN resources describe children’s exposure to domestic violence as potentially traumatic and emphasize supporting children in conjunction with their non-abusive caregivers (National Child Traumatic Stress Network, 2015).

### Parenting self-efficacy as a developmental pathway

Parenting self-efficacy refers to caregivers’ beliefs in their ability to fulfill parenting responsibilities effectively and positively influence their children’s growth and development (Jones and Prinz, 2005; Wittkowski et al., 2017). Grounded in Bandura’s Social Cognitive Theory, parenting self-efficacy reflects caregivers’ confidence in managing parenting challenges, responding to children’s emotional and developmental needs, and fostering supportive parent–child relationships (Bandura, 1997; Jones and Prinz, 2005).

This concept can be further understood through the contemporary coercive control framework, which views domestic violence as an ongoing pattern of domination that may restrict autonomy, decision-making, access to resources, and family relationships rather than as a series of isolated violent incidents (Stark and Hester, 2019). Recent research indicates that interparental coercive control is associated with caregiver psychological distress, parenting difficulties, strained parent–child relationships, and adverse outcomes for children and families (Xyrakis et al., 2024).

Although parenting self-efficacy is related to parenting competence and parenting behaviors, it specifically reflects caregivers’ perceptions of their capabilities rather than their actual parenting skills or observed behaviors (Wittkowski et al., 2017). Higher parenting self-efficacy has been associated with more positive parenting practices and healthier adjustment among parents and children. However, these relationships may be reciprocal and influenced by parent, child, and contextual factors (Glatz et al., 2024; Jones and Prinz, 2005). Parenting self-efficacy therefore provides a valuable framework for examining how caregiver functioning may influence child well-being in families affected by domestic violence.

Domestic violence may undermine parenting self-efficacy by negatively affecting caregivers’ psychological well-being, emotional resources, autonomy, and perceived ability to meet their children’s needs (Levendosky and Graham-Bermann, 2001; Sousa et al., 2022). Within a coercive control framework, abusive partners may restrict caregivers’ decision-making, access to resources, social connections, and authority within the family. These restrictions may weaken caregivers’ sense of control and confidence in fulfilling their parenting responsibilities (Stark and Hester, 2019; Xyrakis et al., 2024).

Caregivers who experience violence may also contend with chronic stress, anxiety, depression, trauma-related symptoms, and fear, which can make it more difficult to maintain emotional availability, consistency, and responsiveness to their children (Sousa et al., 2022). These challenges may increase parenting stress and contribute to uncertainty about how to address children’s emotional, developmental, and behavioral needs (Levendosky and Graham-Bermann, 2001; Sousa et al., 2022).

Diminished parenting self-efficacy may be associated with less effective parenting practices and caregiving conditions that heighten children’s developmental risk. However, these relationships are likely reciprocal and influenced by broader family and social conditions (Glatz et al., 2024; Jones and Prinz, 2005). This proposed pathway highlights caregiver well-being, autonomy, and relational context as important considerations in interventions designed to support children and families affected by domestic violence.

Within this proposed developmental pathway, parenting self-efficacy may serve as a protective resource that supports resilience among families affected by domestic violence. Greater confidence in parenting abilities has been associated with more positive, responsive, and consistent parenting practices. These relationships are reciprocal and influenced by caregiver, child, family, and social conditions (Glatz et al., 2024; Jones and Prinz, 2005). Such caregiving practices may help strengthen children’s emotional security, support parent–child relationships, and promote adaptive coping during periods of family stress (Holt et al., 2008; Sousa et al., 2022).

Conversely, diminished parenting self-efficacy may make it more difficult for caregivers to provide the structure, reassurance, and emotional responsiveness that children need while coping with traumatic experiences. Lower parenting self-efficacy should not, however, be interpreted as an absence of parenting capability, because caregivers may continue to engage in protective and adaptive parenting despite the constraints imposed by domestic violence and coercive control (Sousa et al., 2022; Stark and Hester, 2019). Although parenting self-efficacy alone cannot eliminate the effects of domestic violence, strengthening caregivers’ confidence, autonomy, and capacity may represent a potentially modifiable pathway for promoting child well-being and family resilience that warrants further empirical evaluation (Glatz et al., 2024; Sousa et al., 2022).

### Recommendations for research, practice, and intervention

The effects of domestic violence on children are shaped not only by exposure to violence but also by caregiver functioning, parenting processes, and the availability of supportive relationships and services (Holt et al., 2008; Sousa et al., 2022). In addition to addressing immediate safety, comprehensive responses should include strategies that support caregiver well-being and confidence, strengthen parent–child relationships, and promote children’s developmental well-being. The following recommendations identify priorities for research, clinical and community practice, and family-centered interventions, with particular attention to parenting self-efficacy as a potentially modifiable pathway.

#### Expand qualitative research on family experiences and parenting processes

Qualitative research can provide valuable insights into how domestic violence influences caregiver functioning, parenting self-efficacy, and children’s well-being, complementing information obtained through standardized quantitative measures. For example, qualitative evidence has illuminated children’s perspectives on safety, relationships, coping, agency, and resilience in families affected by domestic violence (Arai et al., 2021). Trauma-informed and developmentally appropriate research approaches may allow caregivers and, when ethically appropriate, children to describe how violence affects parenting confidence, parent–child relationships, coping strategies, and family resilience. These perspectives can inform the development of responsive assessment tools and family-centered interventions that reflect the complex experiences and needs of families affected by domestic violence.

Conducting qualitative research in this area presents important ethical and practical challenges. Caregivers may be reluctant to disclose their experiences because of stigma, concerns about privacy and confidentiality, fear of the person causing harm, or possible consequences following disclosure. Research involving domestic violence therefore requires appropriately trained personnel, trauma-informed interviewing practices, strong confidentiality and safety procedures, and clear plans for responding to participant distress and connecting participants with appropriate services (World Health Organization, 2001, 2016). These protections are especially important when research includes children or when caregivers and children remain in unsafe or coercive environments. Despite these challenges, expanding rigorous qualitative research remains an important priority for developing a more comprehensive understanding of the experiences and processes that may link domestic violence, parenting self-efficacy, and child well-being.

#### Strengthen parenting self-efficacy through family-centered interventions

Interventions for families affected by domestic violence should prioritize immediate safety while also incorporating strategies that support caregiver well-being, strengthen parenting self-efficacy, and promote positive parent–child relationships and secure caregiver-child attachment (Noonan and Pilkington, 2020; Sousa et al., 2022). Trauma-informed programs may include mental health services, parenting support, stress-management strategies, and positive coping approaches to help caregivers respond to their children’s emotional and developmental needs.

Relationship-based interventions may further support secure attachment by strengthening caregiver emotional availability, responsiveness, consistency, and children’s sense of safety within the caregiving relationship (Noonan and Pilkington, 2020). More broadly, parenting interventions have demonstrated benefits for parent–child relationships, caregiver mental health, and children’s emotional and behavioral well-being, although their effectiveness may vary across populations and settings (World Health Organization, 2023). Strengthening caregivers’ confidence and coping capacity may therefore support caregiver functioning, secure attachment, and healthier developmental outcomes for children exposed to domestic violence.

Implementing family-centered interventions requires ongoing investment, interdisciplinary collaboration, and culturally responsive approaches. However, several factors may limit implementation, including restricted funding, stigma surrounding mental health services, workforce shortages, and inconsistent access to appropriate care. Although the evidence base remains heterogeneous, interventions that address the needs of both caregivers and children show promise as a complement to crisis and safety services. They may foster secure attachment, strengthen parent–child relationships, support recovery, and build family resilience (Sousa et al., 2022). Child-focused interventions alone may be insufficient, especially when violence, trauma, or instability persist in the caregiving environment. Coordinated strategies that support both children and their non-abusive caregivers may therefore be particularly important (Spearman et al., 2023). Ongoing evaluation is necessary to identify which intervention components are most effective, for whom, and under what circumstances.

#### Develop trauma-informed and developmentally responsive support for children

Children exposed to domestic violence should have access to trauma-informed and developmentally responsive supports tailored to their individual experiences, strengths, safety, and emotional and behavioral needs. Assessment and intervention planning should consider concerns such as anxiety, depression, trauma-related symptoms, aggression, social withdrawal, low self-esteem, and difficulties with emotional regulation, while recognizing that children vary considerably in their responses to exposure (National Child Traumatic Stress Network, 2015). The type and intensity of support should be informed by the child’s age and developmental level, nature and duration of exposure, family circumstances, current symptoms, and access to protective relationships. Depending on the child’s needs, counseling, caregiver-child interventions, school-based services, peer support, case management, and other evidence-informed resources may strengthen coping and support healthier developmental outcomes. However, intervention effects vary across programs and populations (Latzman et al., 2019).

Timely identification and support depend on coordinated, trauma-informed response and referral systems across healthcare, behavioral health, education, child welfare, domestic violence services, and community-based organizations. Screening or inquiry should be implemented only when trained personnel, confidential procedures, safety protocols, and appropriate referral resources are available. Implementation may be constrained by difficulties engaging children and caregivers, inconsistent identification and referral practices, limited communication across systems, workforce shortages, and insufficient sustained funding. Strengthening interdisciplinary collaboration and continuity of care may improve access to timely and appropriate support, provided that coordination protects family safety, confidentiality, and survivor autonomy (National Child Traumatic Stress Network, 2015).

## Conclusion

Domestic violence remains a significant public health issue with profound implications for children, caregivers, and families. This commentary highlights children’s exposure to domestic violence as an important developmental concern. It proposes parenting self-efficacy as a potential pathway through which domestic violence may influence child well-being. By integrating evidence on caregiver functioning, parenting processes, and children’s developmental outcomes, the commentary underscores the importance of considering family dynamics when designing and evaluating interventions for families affected by domestic violence.

Trauma-informed, family-centered interventions that support parenting self-efficacy may strengthen caregiver functioning, promote supportive parent–child relationships, and improve developmental outcomes for children. Future research should examine parenting self-efficacy as a potentially modifiable developmental pathway and evaluate interventions that address the interconnected needs of caregivers and children. Advancing this line of research may inform policies, clinical practices, and community-based programs that promote family resilience and reduce the long-term consequences of domestic violence for children and families.

## Data Availability

The original contributions presented in the study are included in the article/supplementary material, further inquiries can be directed to the corresponding author.
